# Hyperbaric oxygen therapy in Fournier’s gangrene

**DOI:** 10.1590/S1677-5538.IBJU.2022.0119.1

**Published:** 2022-08-03

**Authors:** José de Bessa

**Affiliations:** 1 Universidade Estadual de Feira de Santana - UEFS Disciplina de Urologia BA Brasil Disciplina de Urologia, Universidade Estadual de Feira de Santana - UEFS, BA, Brasil

## COMMENT

Fournier’s gangrene (FG) is a devastating condition with a high mortality rate. The incidence increases with age and comorbidities, especially Diabetes Mellitus.

Standard treatment consists of aggressive broad-spectrum antibiotics, debridement, and intensive care, with long hospitalization periods and almost always significant sequelae in the eventual survivors.

Adjuvant measures such as Hyperbaric Oxygen Therapy (HBOT) have been offered and used in the treatment of FG, with apparently promising results.

Despite the in vitro biological plausibility and the beneficial effect demonstrated in dogs, the absence of RCTs allows for more appropriate evidence. It limits the recommendations and validity of these findings.

Aware of this limitation, the authors, in a well-conducted paper, summarized the findings of observational studies carried out in the last 25 years on the theme. They demonstrated a significant reduction in the mortality rate in FG patients but could not confirm a reduction in the length of stay and number of debridements (1).

These findings agree with a retrospective review of a large nationwide database of cases of necrotizing soft tissue infection database (45913 subjects) that reported a significant reduction in mortality among those treated after controlling for possible confounders with HBOT (2).

On the other hand, the heterogeneity of the population evaluated; the studies’ observational and retrospective character of the included studies is the main limitation of this metanalysis. These aspects suggest that the described benefits can not have the magnitude observed but signal a scenario of use and cost-effectiveness with HBOT.

Despite some criticism and inspiration by Voltaire: “All is for the best in the best of all worlds.”
